# Interpretable chronic obstructive pulmonary disease identification using chest X-ray radiomics: a multicenter study

**DOI:** 10.1186/s13244-026-02254-z

**Published:** 2026-03-27

**Authors:** Qian Zhou, Weihao Zhai, Taohu Zhou, Yi Wang, Xiuxiu Zhou, Xiaoqing Lin, Jie Li, Huawei Wu, Qi Dai, Yanqing Ma, Fangyi Xu, Hong Zhang, Yanming Ge, Li Fan

**Affiliations:** 1 Department of Radiology, Second Affiliated Hospital of Naval Medical University, Shanghai, China; 2 College of Health Sciences and Engineering, University of Shanghai for Science and Technology, Shanghai, China; 3 Department of Nuclear Medicine, Huadong Hospital Affiliated to Fudan University, Shanghai, China; 4 School of Medical Imaging, Weifang Medical University, Weifang, China; 5 Fujian Provincial Cancer Hospital, Radiation Therapy Center, Fujian, China; 6 Department of Radiology, Union Hospital, Tongji Medical College, Huazhong University of Science and Technology, Wuhan, China; 7 Department of Radiology, Renji Hospital Affiliated to Shanghai Jiao Tong University School of Medicine, Shanghai, China; 8 Department of Radiology, Ningbo No.2 Hospital, Zhejiang, China; 9 Department of Radiology, Zhejiang Provincial People’s Hospital, Zhejiang, China; 10 Department of Radiology, Sir Run Run Shaw Hospital, Zhejiang, China; 11 Department of Radiology, Tianjin Chest Hospital, Tianjin, China; 12 Affiliated Hospital of Weifang Medical University, Weifang, China

**Keywords:** Chronic obstructive pulmonary disease (COPD), Chest X-ray (CXR), Radiomics

## Abstract

**Objectives:**

To construct and validate a combined model integrating chest X-ray (CXR)-based radiomic features and clinical characteristics for chronic obstructive pulmonary disease (COPD) identification, while enhancing model interpretability.

**Materials and methods:**

Paired CXR images and clinical data were collected from 17 hospitals between January 2017 and December 2023. Data from 11 centers were divided into a training cohort and an internal validation cohort at a 7:3 ratio, with data from the remaining 6 centers serving as an external validation cohort. Three models (radiomic model, clinical model, and combined model) were constructed, and the SHapley Additive exPlanations (SHAP) method was used to interpret model performance.

**Results:**

A total of 2433 participants were enrolled, with a mean age of (66.9 ± 11.4) years, including 1564 males and 819 COPD patients. The radiomic model achieved AUCs of 0.760, 0.754, and 0.764 in the training, internal validation, and external validation cohorts, respectively, which were significantly higher than those of the clinical model (AUCs: 0.631, 0.651, and 0.673; all *p* < 0.001). SHAP analysis revealed that age, radiomic features, smoking history, and sex were crucial for COPD identification.

**Conclusions:**

This study successfully constructed a CXR-based combined radiomic-clinical model for COPD, which demonstrated good performance and high accuracy in identifying COPD in this multicenter study. The SHAP method enhanced the model’s interpretability and clinical applicability.

**Critical relevance statement:**

This study develops a CXR radiomic-clinical COPD identification model with SHAP-enhanced interpretability, advancing interpretable, widely applicable COPD screening in clinical radiology.

**Key Points:**

- The clinical screening rate for COPD remains severely inadequate.
- The combined model integrating chest X-ray radiomic features and clinical variables enables accurate differentiation between patients with COPD and non-COPD individuals.
- Global SHAP analysis reveals that radiomic features are the primary factor influencing COPD identification, followed by age, sex, and smoking status.
- Local SHAP analysis can intuitively visualize the model’s decision-making process at the individual sample level.

**Graphical Abstract:**

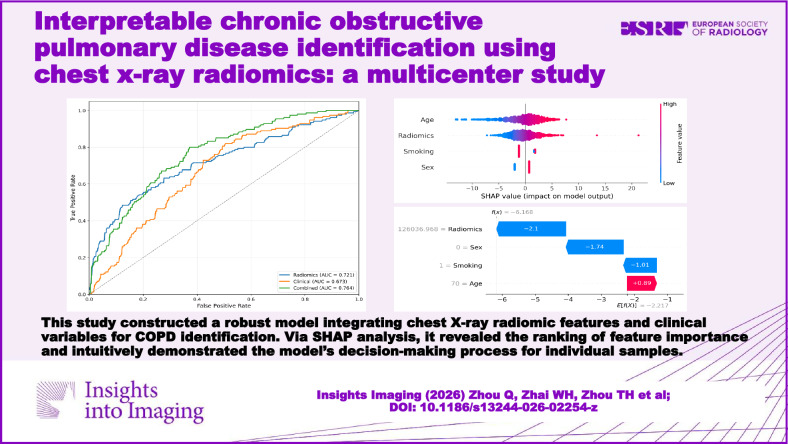

## Introduction

Chronic obstructive pulmonary disease (COPD) is a heterogeneous chronic inflammatory disorder characterized by persistent airflow limitation [1], imposing a heavy economic burden globally [2]. Early screening and intervention are crucial for slowing disease progression [3, 4], but the clinical screening rate for COPD remains severely inadequate. Studies have shown that the pulmonary function test (PFT) rate among residents aged 40 years and above is only 6.7%, with the overall test rate still at a low level [5].

Chest CT can accurately capture the heterogeneous changes in the lung parenchyma of COPD patients [6–8], and deep learning technology has provided tools for identifying potential COPD cases [9]. However, the radiation exposure risk [10] and relatively high costs of CT limit its application in large-scale screening. Chest X-ray (CXR), due to its cost-effectiveness, convenience, and high popularity, is regarded as a potential alternative method for pulmonary function evaluation. Traditional viewpoints hold that the diagnostic value of subjective CXR assessment for COPD is limited [11]. Nevertheless, recent breakthroughs in artificial intelligence (AI) have offered new opportunities [12]. Deep learning models based on CXR can assist in COPD diagnosis by predicting key pulmonary function indicators such as the ratio of forced expiratory volume in 1 s to forced vital capacity (FEV₁/FVC) and the percentage of predicted FEV₁ (FEV₁% pred) [13, 14]. However, deep learning is a “black-box” model with insufficient interpretability, which may affect clinicians’ trust and its clinical application [15].

Radiomics extracts high-dimensional quantitative features (e.g., grayscale, texture complexity, morphology) from medical images in a high-throughput manner, enabling the assessment of pathological and physiological changes from anatomical morphology to the microcosmic level [16]. Currently, most radiomics-related studies on COPD are based on CT [8], while the potential of CXR-based radiomics has not been fully explored. Moreover, CXR is easier to popularize and apply. This study aims to construct a radiomic feature model based on CXR, validate its diagnostic efficacy in distinguishing COPD patients from non-COPD individuals, and introduce the SHapley Additive exPlanations (SHAP) interpretable technique to demonstrate the model’s decision-making logic. Meanwhile, it intends to explore the physiological associations between CXR radiomic features and key pulmonary function indicators (FEV₁/FVC and FEV₁% pred).

## Materials and methods

### Patients and clinical data

**Fig. 1:**
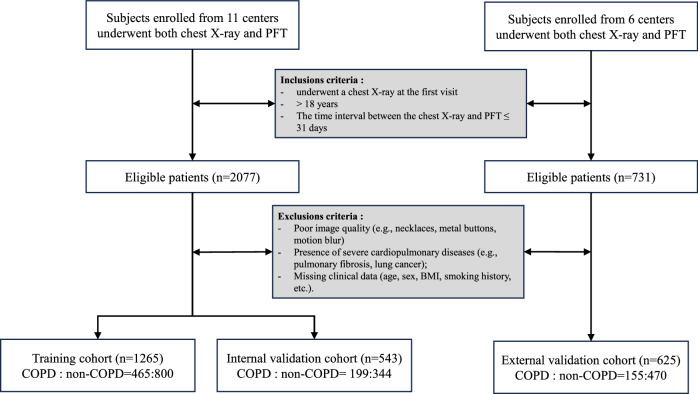
Diagram showing the patient inclusion and exclusion process. COPD, chronic obstructive pulmonary disease; PFT, pulmonary function disease; CT, computed tomography

This was a retrospective study approved by the Ethics Committee (Ethics No. 2024SL043), with informed consent waived due to its retrospective nature. Paired CXR images and clinical data were collected from 17 hospitals between January 2017 and December 2023 (Fig. 1). Clinical information, including age, weight, height, body mass index (BMI), sex, pulmonary function indicators (FEV₁% predicted, FEV₁/FVC), and smoking status, was extracted from the electronic medical record system. The pulmonary function diagnostic criteria for COPD were: FEV₁/FVC < 0.7 and FEV₁ improvement < 200 mL after bronchodilator administration. The non-COPD group was defined as patients with FEV₁/FVC ≥ 0.7 and FEV₁% predicted ≥ 80% after bronchodilator use. Inclusion criteria: ① posteroanterior CXR performed at the first visit; ② age > 18 years; ③ time interval between CXR and PFT ≤ 31 days (to ensure matching of CXR and pulmonary function status). Exclusion criteria: ① substandard images (artifacts from external foreign bodies, severe motion blur, or insufficient exposure); ② concurrent cardiopulmonary diseases that may interfere with lung field assessment (e.g., idiopathic pulmonary fibrosis, lung cancer); ③ missing clinical data (incomplete age, sex, BMI, smoking history, PFT indicators, etc.). Among the 17 centers, data from 11 centers were randomly divided into a training cohort and an internal validation cohort at a 7:3 ratio, while data from the remaining 6 centers served as an independent external validation cohort.

### CXR preprocessing (Fig. 2)

Contrast Limited Adaptive Histogram Equalization (CLAHE) was applied to enhance the contrast of lung field regions and suppress background noise [17]. Linear grayscale normalization was then performed to map pixel values to the range of 0–1, eliminating grayscale differences between devices.

**Fig. 2:**
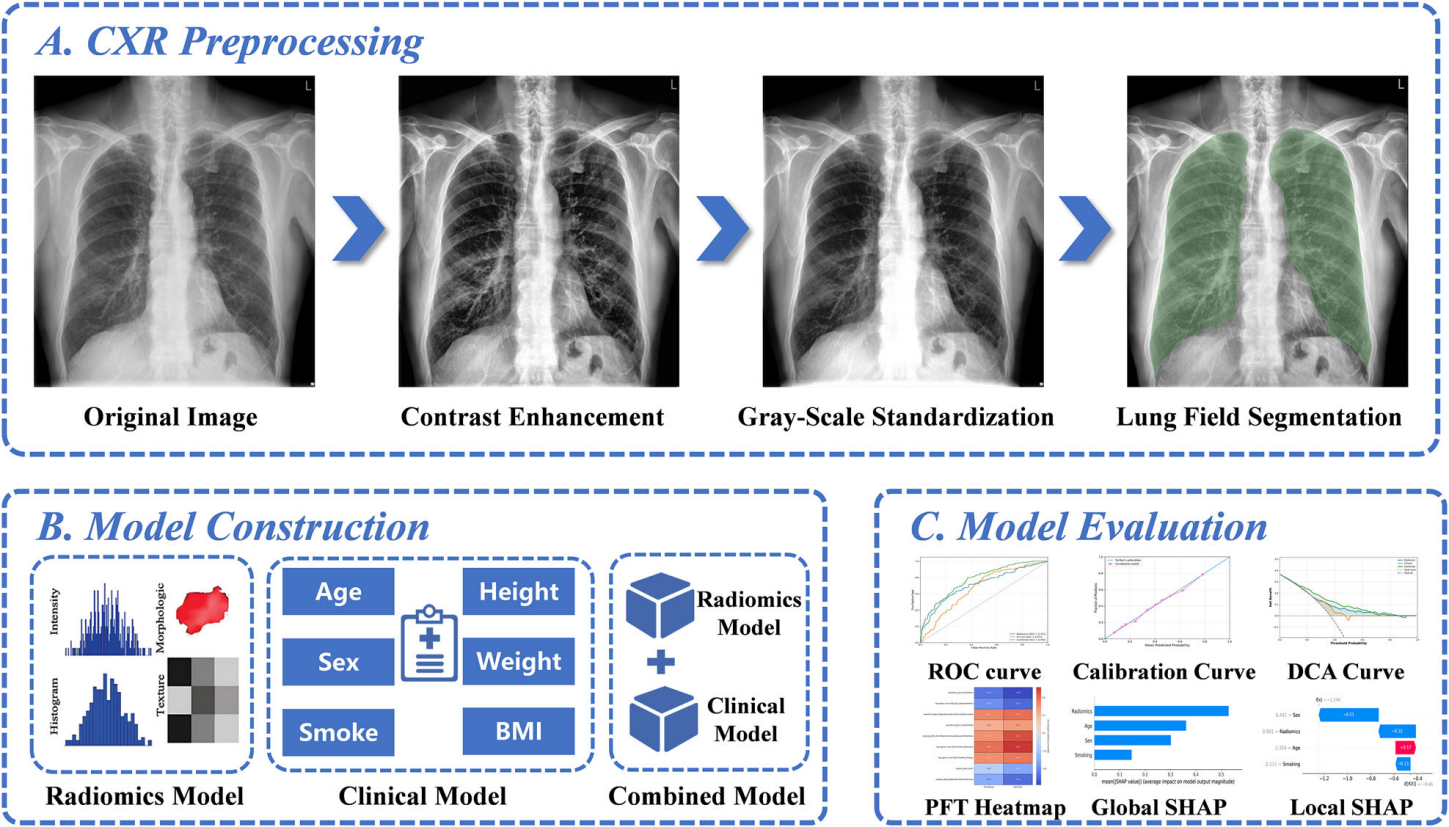
Study workflow overview. **A** CXR preprocessing: The original chest X-ray image, after undergoing contrast enhancement (Contrast Limited Adaptive Histogram Equalization, CLAHE) and grayscale normalization to the [0,1] range, is automatically segmented into bilateral lung fields using the nnU-Net model. **B** Model construction: Three models were constructed: radiomics, clinical, and combined. **C** Model evaluation: Model performance evaluation was conducted using ROC curves, calibration curves, and DCA curves. The biological interpretability was validated through correlation analysis between radiomic features and pulmonary function indices (FEV₁%pred and FEV₁/FVC). The decision logic of the model was revealed using SHAP’s global and local interpretation mechanisms

### CXR segmentation

Bilateral lung field regions of interest (ROIs) were automatically segmented using the pre-trained nnU-Net deep learning framework (trained on manually segmented lung fields) [18]. To validate segmentation consistency, two radiologists (T.H.Z., 5 years of chest image interpretation experience; Y.W., 3 years) manually segmented the same set of 100 COPD and 100 non-COPD cases, with the Dice Similarity Coefficient (DSC) calculated to assess inter-radiologist agreement.

A total of 200 cases manually segmented by the two radiologists (50 COPD and 50 non-COPD cases per radiologist without overlapping cases) were randomly selected, and the DSC was calculated by comparing these manual segmentation results with their corresponding automatic segmentation results.

### Radiomic feature extraction and selection

A total of 558 pulmonary radiomic features were extracted from each ROI using the open-source software package PyRadiomics (Version 3.0.1, official website: https://pyradiomics.readthedocs.io/en/latest/), including first-order statistical features (overall distribution of pixel intensity), shape features (spatial morphology of lung fields), and texture features (spatial heterogeneity between pixels). After Z-score standardization to eliminate differences in numerical scales, a multi-stage selection strategy was adopted for dimensionality reduction: ① Pearson correlation coefficients between features were calculated, and highly redundant features with a correlation coefficient > 0.9 were eliminated; ② the minimum redundancy maximum relevance (mRMR) algorithm [19] was applied to select the top 20 features highly correlated with COPD and low in redundancy; ③ finally, the least absolute shrinkage and selection operator (LASSO) regression algorithm combined with 10-fold cross-validation was used to determine the final parameters and features.

### Model construction

Seven machine learning methods (logistic regression (LR), naive Bayes (NB), multi-layer perceptron (MLP), random forest (RF), support vector machine (SVM), XGBoost, and Ridge classifier) were tested on the training, internal validation, and external validation cohorts. The method with the best performance was selected to construct three types of models: clinical model, radiomic model, and combined model. Clinically significant risk variables were screened via univariate logistic regression analysis, followed by multivariate logistic regression analysis to establish the clinical model. The radiomic model was constructed using LR with the selected radiomic features as inputs. The combined model was built by integrating clinical variables and radiomic features, fusing the two types of information through multivariate logistic regression.

### Model evaluation

The receiver operating characteristic (ROC) curve and area under the curve (AUC) were used to evaluate the model’s ability to distinguish COPD from non-COPD patients. Calibration curves (Hosmer-Lemeshow test) were employed to assess the calibration degree between the model predictions and actual outcomes. Decision curve analysis (DCA) was performed to evaluate the clinical utility of the models. Subgroup analysis of the combined model stratified by gender and age (with a cutoff of 65 years) was conducted to evaluate the model stability across different populations in both internal and external validation cohorts.

Spearman’s rank correlation analysis was used to quantify the strength of association between radiomics features and FEV₁%pred as well as FEV₁/FVC, thereby revealing the correlation between radiomics features and functional impairment.

For the radiomic model, SHAP summary plots and beeswarm plots were used to interpret feature importance and the model’s decision-making mechanism. For the combined model, the SHAP method was applied to quantify the contribution of each variable to the identification results, achieving global interpretation at the feature level (revealing core driving factors and biological mechanisms at the population level) and local interpretation at the individual level (illustrating the direction and intensity of feature effects in a single sample).

### Statistical analysis

Statistical analyses were performed using R software (version 4.2.2; http://www.rproject.org) and Python (version 3.10.11; https://www.python.org/). Quantitative data are presented as mean ± standard deviation. Independent samples *t*-test was used for normally distributed continuous variables, Mann–Whitney U test for non-normally distributed continuous variables, and chi-square test for categorical variables. Multivariate logistic regression analysis was used to screen independent predictors from clinical variables, with a statistical significance threshold of *p* < 0.05. LASSO regression analysis was performed using the “glmnet” package. DeLong’s test was used to compare the areas under the curve (AUCs) of the clinical model, radiomics model, and combined model. Calibration curves and multivariate logistic regression analyses were generated using the “rms” package. Decision curve analysis (DCA) was conducted using the “rmda” package. SHAP plots were generated using the SHAP library for interpretability analysis.

## Results

### Clinical characteristics

A total of 2433 patients were enrolled, including 1564 males and 869 females (1614 in the non-COPD group and 819 in the COPD group), with a mean age of (66.9 ± 11.4) years. Baseline demographic characteristics are presented in Table 1. A total of 2433 CXR images were collected, and detailed CXR acquisition parameters are provided in Table S1. Samples from 11 centers were divided into the training cohort and internal validation cohort at a 7:3 ratio. The patient distribution across these 11 centers was as follows: Hospital 1 (*n* = 1353), Hospital 2 (*n* = 37), Hospital 3 (*n* = 37), Hospital 4 (*n* = 14), Hospital 5 (*n* = 65), Hospital 6 (*n* = 15), Hospital 7 (*n* = 50), Hospital 8 (*n* = 44), Hospital 9 (*n* = 30), Hospital 10 (*n* = 67), and Hospital 11 (*n* = 37). The training cohort consisted of 800 non-COPD patients and 465 COPD patients, while the internal validation cohort included 344 non-COPD patients and 199 COPD patients. The external validation cohort was composed of data from Hospital 12 (*n* = 488), Hospital 13 (*n* = 3), Hospital 14 (*n* = 5), Hospital 15 (*n* = 77), Hospital 16 (*n* = 16), and Hospital 17 (*n* = 96), including 470 non-COPD patients and 155 COPD patients. In all cohorts, significant differences were observed between the non-COPD and COPD groups in terms of age, sex, height, weight, BMI, smoking status, and pulmonary function indicators (FEV₁% predicted, FEV₁/FVC) (all *p* < 0.05).

**Table 1:** Baseline characteristics of the study population

Characteristic	Training cohort (*n* = 1265)			Internal validation cohort (*n* = 543)			External validation cohort (*n* = 625)		
	COPD (*n* = 465)	Non-COPD (*n* = 800)	*p*-value	COPD (*n* = 199)	Non-COPD (*n* = 344)	*p*-value	COPD (*n* = 155)	Non-COPD (*n* = 470)	*p*-value
Age	68.5 ± 9.5	62.6 ± 12.2	< 0.001	68.2 ± 9.6	62.4 ± 12.3	< 0.001	69.4 ± 7.2	64.1 ± 11.4	< 0.001
Sex			< 0.001			< 0.001			< 0.001
Male	387 (83.2%)	446 (55.8%)		161 (80.9%)	209 (60.8%)		123 (79.4%)	238 (50.6%)	
Female	78 (16.8%)	354 (44.2%)		38 (19.1%)	135 (39.2%)		32 (20.6%)	232 (49.4%)	
Height	1.66 ± 0.07	1.62 ± 0.08	< 0.001	1.65 ± 0.06	1.63 ± 0.08	< 0.001	1.65 ± 0.07	1.61 ± 0.08	< 0.001
Weight	64.5 ± 9.5	66.1 ± 10.8	0.040	66.1 ± 10.2	63.1 ± 8.7	< 0.001	64.9 ± 9.2	66.8 ± 11.2	0.036
BMI	23.5 ± 2.8	25.1 ± 3.2	< 0.001	23.1 ± 2.7	24.9 ± 3.1	< 0.001	24.0 ± 2.7	25.6 ± 3.6	< 0.001
Smoking			< 0.001			< 0.001			< 0.001
Non-smoker	254 (54.6%)	590 (73.8%)		114 (57.3%)	258 (75.0%)		91 (58.7%)	365 (77.7%)	
Former smoker	82 (17.6%)	67 (8.4%)		25 (12.6%)	28 (8.1%)		22 (14.2%)	33 (7.0%)	
Current smoker	129 (27.7%)	143 (17.9%)		60 (30.2%)	58 (16.9%)		42 (27.1%)	72 (15.3%)	

*BMI* body mass index

### Consistency assessment between manual and automatic segmentation

The inter-radiologist DSC was 0.947 (95% CI: 0.946–0.950). The overall DSC between automatic and manual segmentation reached 0.951 (95% CI: 0.947–0.956), with a DSC of 0.956 (95% CI: 0.950–0.961) for COPD cases and 0.947 (95% CI: 0.940–0.954) for non-COPD cases, indicating high segmentation reliability.

### Feature selection and radiomics signature construction

A total of 558 radiomic features were standardized using Z-score. After Pearson correlation analysis, 499 redundant features were eliminated, leaving 59 features. Finally, 9 radiomic features with non-zero coefficients were selected via LASSO regression (Fig. S1).

### Performance comparison of radiomic, clinical, and combined models

**Fig. 3:**
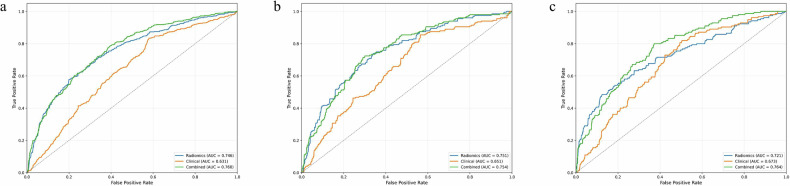
Diagnostic performance of the clinical factors model, radiomics signature, and combined model was assessed and compared through ROC curves in the training (**a**), internal validation (**b**) and external validation (**c**) cohorts. ROC, receiver operating characteristics; AUC, area under the receiver operating characteristic curve

Table 2 shows that among the 7 machine learning methods, LR exhibited the most stable performance, with AUCs of 0.760 (95% CI: 0.733–0.786), 0.754 (95% CI: 0.711–0.794), and 0.764 (95% CI: 0.724–0.804) in the training, internal validation, and external validation cohorts, respectively. The performance of the radiomic, clinical, and combined models constructed based on LR is presented in Table 3 and Fig. 3. The radiomic model showed good discriminative ability, with AUCs of 0.746 (95% CI: 0.717–0.775), 0.751 (95% CI: 0.709–0.791), and 0.721 (95% CI: 0.671–0.767) in the three cohorts. The clinical model included independent predictors (age, sex, smoking status) identified by multivariate regression analysis (Table 4). The combined model was constructed by integrating radiomic features and clinical variables.

**Table 2:** Comparison of the performance of seven different machine learning methods for combined models in the external validation cohort

Model		AUC (95% CI)	Accuracy (%)	Sensitivity (%)	Specificity (%)	PPV (%)	NPV (%)
Training cohort	Logistic Regression	0.760 (0.733–0.786)	59.1	90.1	41.1	47.1	87.7
	Random Forest	0.877 (0.857–0.897)	65.2	94.4	48.3	51.5	93.7
	Naive Bayes	0.730 (0.701–0.758)	62.1	80.4	51.4	49.0	81.9
	MLP Neural Network	0.835 (0.812–0.858)	63.9	91.8	47.6	50.5	90.9
	SVM	0.855 (0.833–0.878)	74.4	84.3	68.6	61.0	88.3
	XGBoost	0.937 (0.923–0.950)	80.6	94.2	72.6	66.7	95.6
	Ridge Classifier	0.797 (0.771–0.822)	68.5	81.1	61.1	54.8	84.7
Internal validation	Logistic Regression	0.754 (0.711–0.794)	58.7	89.4	41.0	46.7	87.0
	Random Forest	0.813 (0.775–0.849)	74.4	87.4	46.8	48.7	86.6
	Naive Bayes	0.718 (0.674–0.761)	60.6	79.9	49.4	47.7	81.0
	MLP Neural Network	0.803 (0.762–0.839)	63.9	89.4	49.1	50.4	88.9
	SVM	0.821 (0.784–0.859)	69.1	82.4	61.3	55.2	85.8
	XGBoost	0.831 (0.794–0.866)	70.3	80.9	64.2	56.7	85.3
	Ridge Classifier	0.797 (0.757–0.835)	68.3	79.9	61.6	54.6	84.1
External validation	Logistic Regression	0.764 (0.724–0.804)	67.0	80.0	62.8	41.5	90.5
	Random Forest	0.702 (0.660–0.747)	66.1	63.2	67.0	38.7	84.7
	Naive Bayes	0.732 (0.689–0.779)	73.3	52.9	80.0	46.6	83.7
	MLP Neural Network	0.687 (0.643–0.733)	58.4	75.5	52.8	34.5	86.7
	SVM	0.722 (0.681–0.765)	68.2	69.7	67.7	41.5	87.1
	XGBoost	0.729 (0.690–0.771)	64.0	72.3	61.3	38.1	87.0
	Ridge Classifier	0.688 (0.637–0.738)	74.7	49.0	83.2	49.0	83.2

**Table 3:** Comparison of diagnostic performance of the radiomic model, clinical model, and combined model in the training and internal and external validation cohorts

Model	AUC (95% CI)	Accuracy (%)	Sensitivity (%)	Specificity (%)	PPV (%)	NPV (%)	*p*-value of DeLong test	
							vs. radiomics model	vs. combined model
Radiomic model								
Training cohort	0.746 (0.717–0.775)	69.4	63.9	72.6	57.6	77.6	-	-
Internal validation cohort	0.751 (0.709–0.791)	70.2	64.8	73.3	58.4	78.3	-	-
External validation cohort	0.721 (0.671–0.767)	77.9	48.4	87.7	56.4	83.7	-	-
Clinical model								
Training cohort	0.631 (0.600–0.661)	57.2	83.4	42.0	45.5	81.4	< 0.001	< 0.001
Internal validation cohort	0.651 (0.604–0.699)	58.6	85.4	43.0	46.4	83.6	< 0.001	< 0.001
External validation cohort	0.673 (0.628–0.720)	57.6	81.9	49.6	34.9	89.3	0.073	< 0.001
Combined model								
Training cohort	0.760 (0.733–0.786)	59.1	90.1	41.1	47.1	87.7	0.255	-
Internal validation cohort	0.754 (0.711–0.794)	58.7	89.4	41.0	46.7	87.0	0.868	-
External validation cohort	0.764 (0.724–0.804)	67.0	80.0	62.8	41.5	90.5	0.024	-

**Table 4:** Univariable and multivariable logistic regression analysis

Variable	Univariable analysis		Multivariable analysis	
	OR [95% CI]	*p*-value	OR [95% CI]	*p*-value
Age	1.05 (1.04–1.06)	< 0.001	1.04 (1.03–1.06)	< 0.001
Sex	3.52 (2.76–4.49)	< 0.001	2.24 (1.56–3.22)	< 0.001
Height	1.06 (1.05–1.08)	< 0.001	1.06 (0.93–1.21)	0.3666
Weight	0.98 (0.97–0.99)	< 0.001	0.96 (0.82–1.13)	0.6609
BMI	0.83 (0.80–0.86)	< 0.001	0.91 (0.59–1.40)	0.6648
Smoking	1.34 (1.18–1.51)	< 0.001	1.47 (1.02–2.12)	0.0368

*BMI* body mass index, *OR* odds ratio, *CI* confidence interval

DeLong test results revealed significant differences in AUC between the combined model and the clinical model across all three cohorts (all *p* < 0.001). A significant difference in AUC was observed between the combined model and the radiomic model in the external validation cohort (0.764 vs. 0.721, *p* = 0.024), but no significant differences were found in the training cohort (0.760 vs. 0.746, *p* = 0.255) or internal validation cohort (0.754 vs. 0.751, *p* = 0.868).

**Fig. 4:**
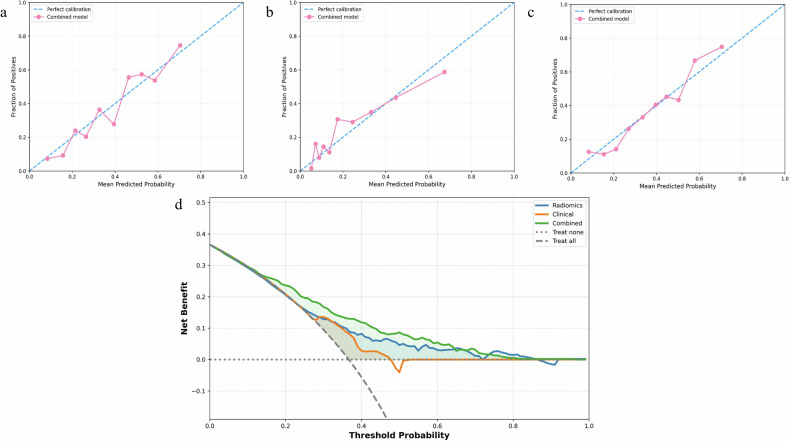
Calibration curves and DCA curves. The combined model calibration curves in training (**a**), internal validation (**b**), and external validation (**c**) cohorts. Calibration curves indicate the goodness-of-fit of the model. **d** Decision curve analysis for different models

Hosmer-Lemeshow goodness-of-fit test (Fig. 4a–c) indicated that the calibration curves of the combined model for COPD identification were highly consistent with actual data in all cohorts (*p*-values: 0.898, 0.557, and 0.903, respectively). Decision curve analysis (DCA) (Fig. 4d) showed that in the training cohort, when the risk probability threshold for clinical decision-making exceeded 0.2, the combined model yielded higher clinical net benefit than the clinical model alone.

### Subgroup analysis

Fig. S2 shows the following results: for the internal validation cohort, the AUC was 0.705 in the female subgroup, 0.695 in the male subgroup, 0.721 in the subgroup aged > 65 years, and 0.770 in the subgroup aged ≤ 65 years; for the external validation cohort, the AUC was 0.696 in the female subgroup, 0.700 in the male subgroup, 0.725 in the subgroup aged > 65 years, and 0.789 in the subgroup aged ≤ 65 years.

### Correlation between radiomic features and pulmonary function indicators

**Fig. 5:**
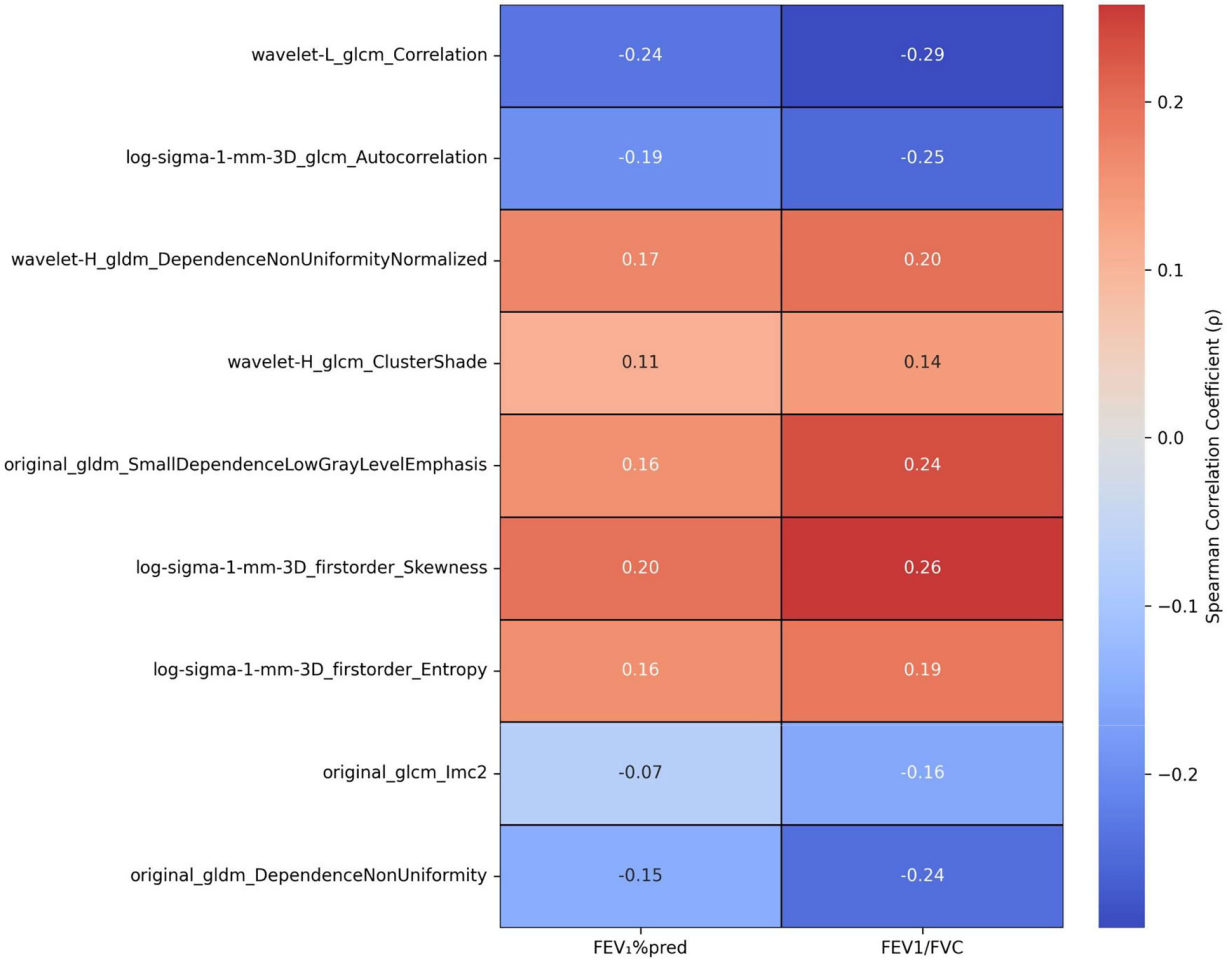
The heatmap illustrates the correlation coefficients between radiomic features and pulmonary function indices (FEV₁%pred and FEV_1_/FVC). The color gradients in the heatmap represent the strength and direction of these correlations

As shown in the correlation heatmap (Fig. 5), all radiomics features exhibited a weak association with FEV₁%pred and FEV₁/FVC (|ρ| < 0.3). Regarding the association with FEV₁/FVC, log-sigma-1-mm-3D_firstorder_Skewness showed the strongest positive correlation (ρ = 0.26), while wavelet-L_glcm_Correlation had the strongest negative correlation (ρ = −0.29). For the association with FEV₁%pred, these two features also ranked as the strongest positive (ρ = 0.20) and negative (ρ = −0.24) correlates, respectively.

### Model interpretability

In the radiomic model, SHAP summary plots and beeswarm plots (Fig. S1c–d) indicated that original_gldm_DependenceNonUniformity was the most critical feature for COPD identification, with the widest distribution and highest dispersion of SHAP values; the importance of other features decreased sequentially.

**Fig. 6:**
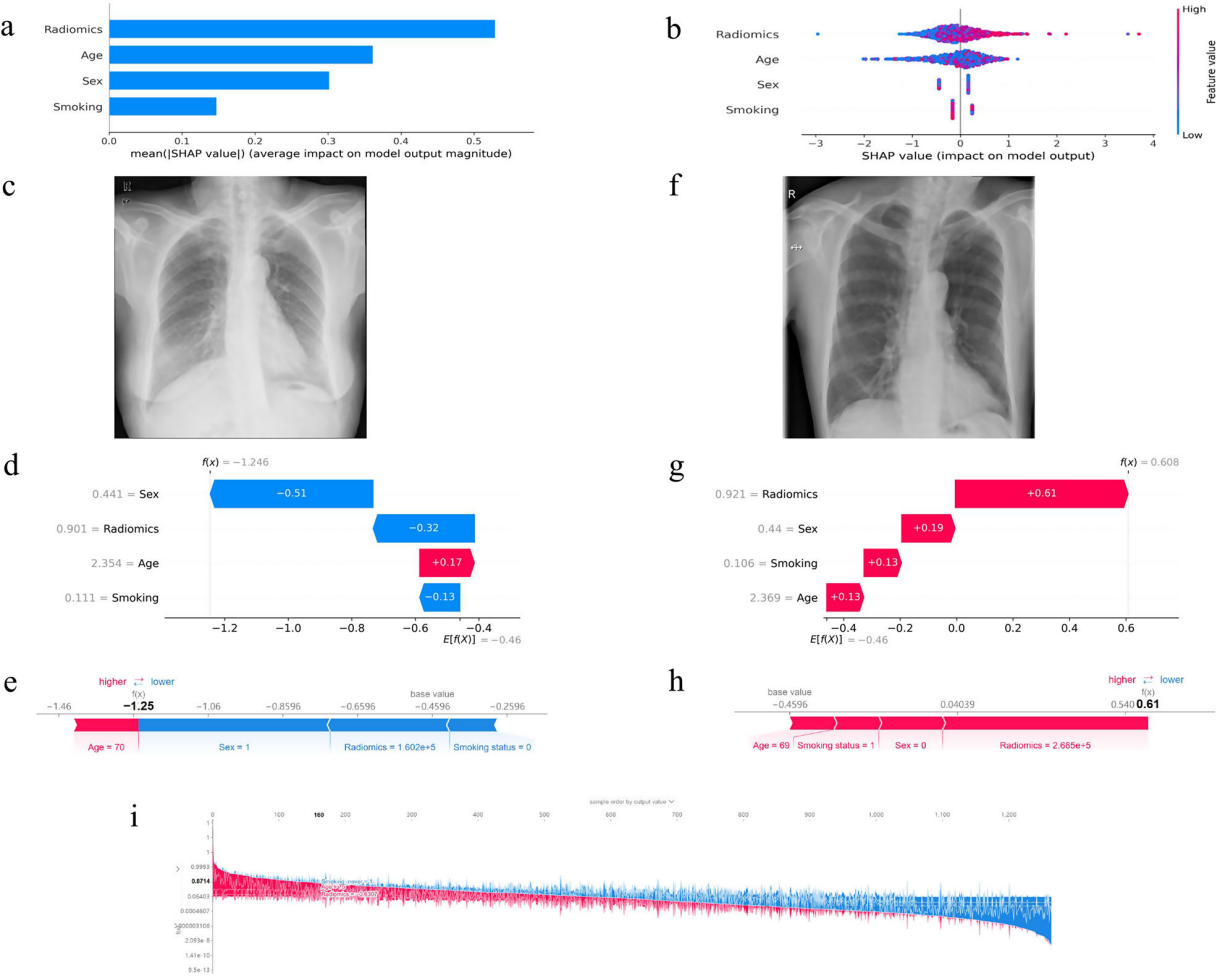
Global and local model explanation by the SHAP method. **a** SHAP bar plot showing the mean absolute SHAP values for each feature of combined model, indicating the overall importance of features in predicting COPD. **b** SHAP beeswarm plot illustrating the distribution of SHAP values for each feature of combined model. The color gradient represents the feature values (red for high values and blue for low values). **c** Chest X-ray of a 70-year-old never-smoking female. **d**, **e** Waterfall plots and force plots show the contribution of each feature to the identification in an individual at low risk of COPD. **f** Chest X-ray of a 69-year-old male who has quit smoking for over a year. **g**, **h** Waterfall plots and force plots show the contribution of each feature to the identification in an individual at high risk of COPD. The base value (gray vertical line) represents the average output of the model, while the arrows indicate how each feature drives the identification result [f(*x*)] toward either the COPD (red) or non-COPD (blue) class. **i** Force plot for the training cohort. Each point on the *x*-axis represents a patient, and the magnitude of feature contributions is indicated by red (onset) and blue (non-onset) segments: a larger red portion for an individual indicates a higher probability of COPD

In the combined model, global and local interpretations were achieved using the SHAP method. For global interpretation (Fig. 6a, b), SHAP summary plot (Fig. 6a) showed that radiomic features had the highest mean absolute SHAP values, exerting the greatest impact on model output, followed by age, sex, and smoking status. SHAP beeswarm plot (Fig. 6b) revealed that radiomic features and age had widely distributed SHAP values, with high radiomic feature values and old age corresponding to positive SHAP values, which positively promoted COPD identification; smoking status and sex (categorical variables) had relatively concentrated SHAP values, exhibiting specific contribution patterns.

For local interpretation (Fig. 6c–i), waterfall plots and force plots illustrated one case identified as non-COPD (FEV₁% predicted: 76.2%, FEV₁/FVC: 76.2%). The individual contributions of each feature are presented in the corresponding waterfall plot and force plot (Fig. 6d, e): radiomic features, female sex, and never-smoking status all contributed to “non-COPD” identification, while old age had no such effect. Another case identified as COPD (FEV₁% predicted: 48.1%, FEV₁/FVC: 50.4%) was shown in Fig. 6g, h: all features, including radiomic features, old age, and smoking status, promoted COPD identification. Figure 6i is the force plot for COPD identification in the training cohort, where the longer the red line corresponding to a patient, the higher the probability of COPD identification.

## Discussion

This study constructed a combined model integrating radiomics and clinical factors and developed an interpretable non-invasive screening tool via the SHAP method. External validation showed that the combined model achieved a significantly higher AUC than the clinical and radiomics models alone, with stable performance across all subgroups. Characterized by high sensitivity and NPV but moderate accuracy, specificity and PPV, the model is more suitable for preliminary screening scenarios, as it can accurately identify affected individuals, rule out negative cases and reduce the risk of missed diagnosis.

SHAP analysis revealed that among clinical variables, age, sex, and smoking history were key indicators for COPD identification, which is consistent with previous research findings [8]. This may be related to the epidemiological distribution characteristics of COPD and airway damage caused by smoking [20]. The top 3 radiomic features were original_gldm_DependenceNonUniformity, log-sigma-1-mm-3D_firstorder_Entropy, and original_glcm_Imc2, all of which reflect the uneven grayscale distribution in lung fields. This phenomenon may be attributed to the fact that radiomics reflects grayscale and textural features on CXR, which may indirectly indicate pathological changes such as lung marking alterations, emphysema, or hyperinflation. Correlation analysis showed that all radiomics features had a weak association with lung function indices, which may be due to the fact that lung function is regulated by factors such as airway structure, pulmonary vascular status, and ventilation-perfusion matching, while radiomics features only reflect partial changes in lung microstructure and cannot fully cover the complex pathophysiological mechanisms underlying lung function.

The accessibility and applicability of COPD screening methods restrict the effectiveness of prevention and treatment. The study has shown that less than 10% of COPD patients have undergone PFT, and the definitive diagnosis rate is less than 1% [21]. Although chest CT has certain value in COPD identification, staging, and classification [7, 22], its application in large-scale COPD screening is limited due to radiation exposure and high inspection costs. In contrast, CXR is more widely available with lower cost and radiation exposure, making it suitable for COPD preliminary screening in health check-ups and resource-limited settings, which helps improve the early diagnosis rate.

CXR screening for COPD has been extensively investigated, as summarized in Table S2. A case-control study confirmed differences in diaphragmatic movement during forced breathing between COPD and non-COPD populations, but this indicator relies on dynamic CXR and is not suitable for routine use [23]. Another case-control study developed a diagnostic model combining quantitative CXR parameters and clinical factors; while its specificity reached 100%, the sensitivity was only 10%, limiting clinical utility [11]. Existing deep learning models have a “black-box” limitation. For instance, a single-center retrospective study achieved a high AUC in predicting FEV₁/FVC, but failed to explain the calculation process of pulmonary function indicators, making it difficult for clinicians to assess its credibility [24]. This study established a clinically translatable COPD screening method based on routine posteroanterior CXR, with a large-scale multicenter PFT-matched dataset for generalizability, pixel-level pathological quantification via radiomics features, and the integration of the SHAP technique to enhance model interpretability.

This study also has certain limitations: first, as a retrospective design, it inevitably has selection bias; second, the presence of physiological structures such as ribs in CXR may potentially interfere with the extraction of radiomic features and model performance [25], and we will explore the model performance after rib suppression in future research; finally, only basic clinical information such as age, sex, and smoking status was included, while potential COPD-related clinical risk factors such as comorbidities and family history were not incorporated, which may have limited the model performance.

In conclusion, the diagnostic efficacy of the combined radiomic-clinical feature model constructed in this study is significantly superior to that of the clinical model alone. The interpretability achieved through SHAP analysis not only enhances its clinical applicability but also provides support for its integration into routine clinical practice. Future prospective validation and model optimization are needed to confirm its efficacy and universality in different patient populations.

## Supplementary information

ELECTRONIC SUPPLEMENTARY MATERIAL

## Data Availability

The datasets generated or analyzed during this study are available from the corresponding author upon reasonable request.

## Abbreviations

AUC: Area under the curve
COPD: Chronic obstructive pulmonary disease
CXR: Chest X-ray
DCA: Decision curve analysis
FEV_1_/FVC: Ratio of forced expiratory volume in 1 s to forced vital capacity
FEV_1_% predicted: Percentage of predicted FEV_1_
LASSO: Least absolute shrinkage and selection operator
PFT: Pulmonary function test
ROC: Receiver operating characteristic
ROI: Region of interest
SHAP: SHapley Additive exPlanations
