# Supplementary material for: Interpretable chronic obstructive pulmonary disease identification using chest X-ray radiomics: a multicenter study
Source: Insights Imaging. 2026 Mar 27;17:81. doi: 10.1186/s13244-026-02254-z (PMC13031562; doi:10.1186/s13244-026-02254-z)
Supplement: Supplementary file 1 — ELECTRONIC SUPPLEMENTARY MATERIAL [file 13244_2026_2254_MOESM1_ESM.docx]

**Supplementary Material**

**Supplementary table**

**Table S1 Acquisition protocols for different chest radiography stands**

| **Equipment** | **Philips Digital Diagnost** | **GE Optima XR646 HD** | **SIEMENS FD-X** | **FUJIFILM Medical Systems (model unspecified)** | **Agfa (model unspecified)** | **UIH uDR** |
| --- | --- | --- | --- | --- | --- | --- |
| Tube voltage(kV) | 80 | 85 | 90 | 80 | 70 | 80 |
| Milliampere-second(mAs) | AEC | AEC | AEC | AEC | AEC | AEC |
| Focal Spot Size(mm) | 2 | 1.3 | 1.2 | 1.2 | 1.2 | 1.2 |
| Source-to-Image Receptor Distance(cm) | 180 | 180 | 180 | 180 | 180 | 180 |

| **Equipment** | **Canon AXIOM-Multix M** | **KODAK DR 7500** | **Mindray DigiEye** | **Carestream Health (model unspecified)** | **TCL MEDICAL SYSTEMS (model unspecified)** | **SHIMADZU RAD speed** |
| --- | --- | --- | --- | --- | --- | --- |
| Tube voltage(kV) | 105 | 110 | 110 | 125 | 80 | 110 |
| Milliampere-second(mAs) | AEC | AEC | AEC | AEC | AEC | AEC |
| Focal Spot Size(mm) | 2 | 1.2 | 1 | 1.2 | 2 | 2 |
| Source-to-Image Receptor Distance(cm) | 180 | 180 | 180 | 180 | 180 | 180 |

***AEC*** dose modulation with automatic exposure control.

**Table S2 Comparison of Performance and Research Design of Chest Radiograph-Based COPD Screening Models**

| **Study** | **Research Design** | **Technical Approach** | **Key Performance Metrics** | **Limitations** |
| --- | --- | --- | --- | --- |
| Hida et al [23], 2019 | Single-center, retrospective case-control | Dynamic chest radiography; time-resolved quantitative analysis; diaphragmatic excursion/peak motion speed measurement | Diaphragmatic motion parameter comparison; correlation with pulmonary function | Single-center, small sample size; dynamic CXR-dependent (not for routine screening) |
| Harder et al [11], 2017 | Nested case-control (1:1) | CXR quantitative + qualitative features | PPV=100%, Sensitivity=10%, AUC=0.811 | extremely low sensitivity; no multicenter validation |
| Schroeder et al [24], 2019 | Single-center retrospective | Pretrained ResNet18 CNN | AUC = 0.814±0.005,  Sensitivity 0.630±0.040, Specificity  0.832±0.025 | Single-center; black-box CNN; no external validation |
| Our study | Multicenter Retrospective Study | CXR radiomics +SHAP | External validation set: AUC=0.764, Accuracy 67.0%, Sensitivity 80.0%, Specificity 62.8%, PPV 41.5%, NPV 90.5% | moderate performance in terms of accuracy, specificity, and PPV |

**Supplementary figure**


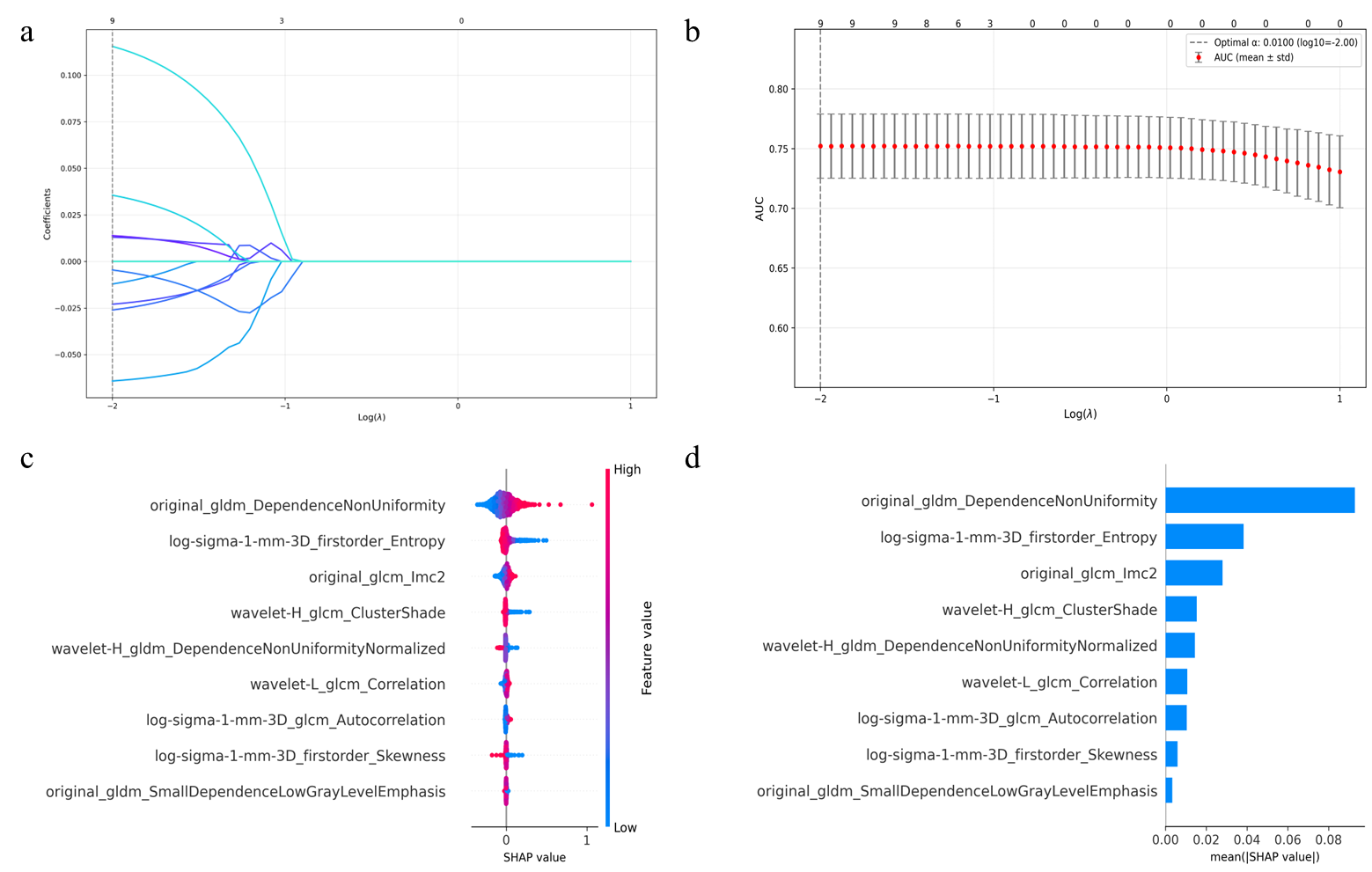


**Fig. S1 LASSO coeffcient of radiomic features.** (**a)** The LASSO coefficient profiles of the 59 radiomics features. A vertical line Was generated at the log (λ) value by using tenfold cross-validation, where the optimal λ value resulted in 9 radiomics features. The optimal λ value of 0.01 was selected. The X-axis on the top indicates the number of nonzero coeffcient features in the model. (**b)** The black vertical line was drawn at the value selected using tenfold cross-validation in (a). The X-axis on the top indicates the number of nonzero coeffcient features in the model. (**c)** SHAP beeswarm plot illustrating the distribution of SHAP values for each radiomic feature. Y-axis lists features ranked by importance, x-axis represents SHAP values (impact on model output), and the color gradient indicates feature values. (**d)** SHAP bar plot showing the mean absolute SHAP values of 9 radiomic features, ranked by importance. *LASSO* least absolute shrinkage and selection operator


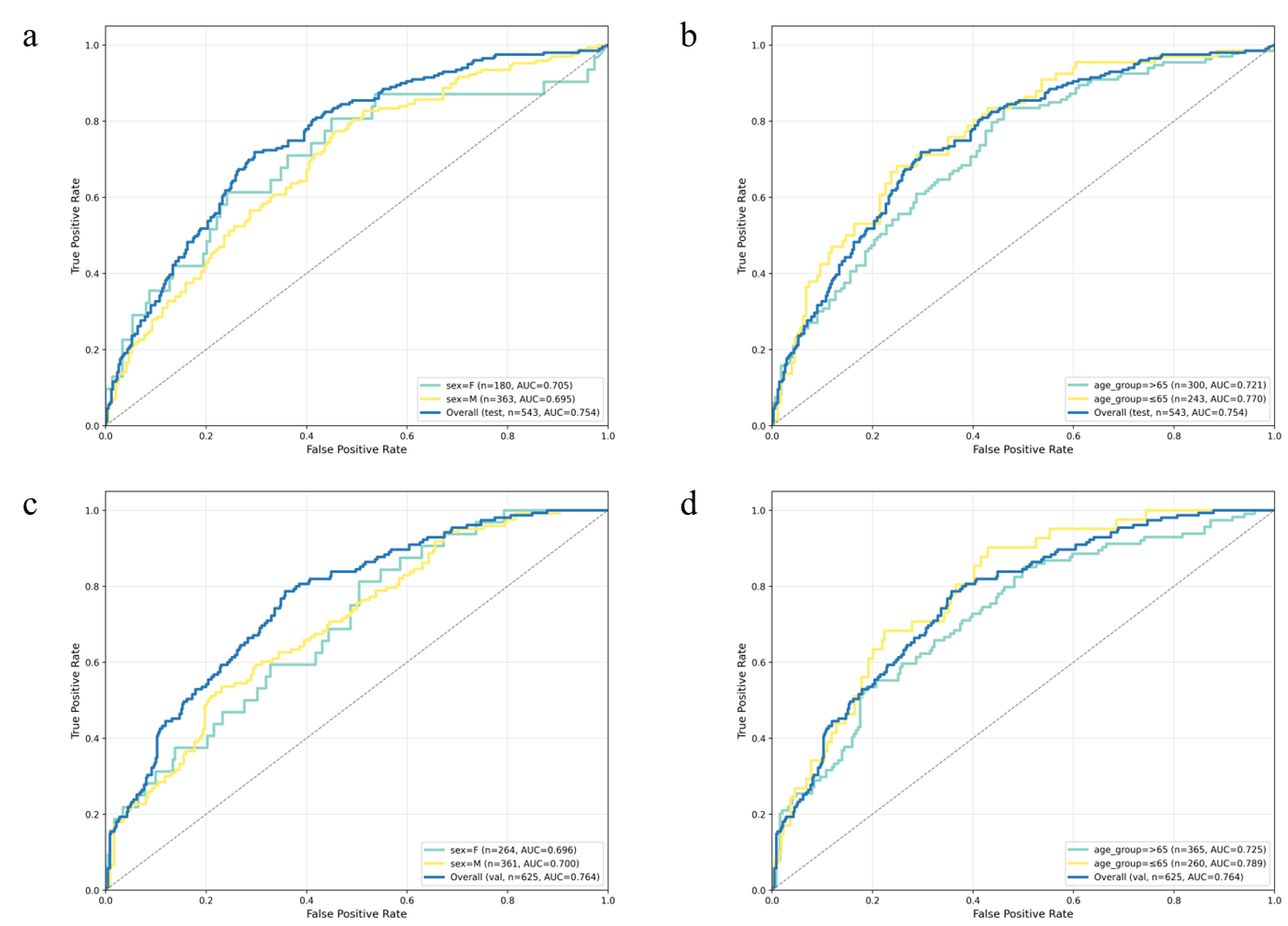


**Fig. S2 Subgroup analyses stratified by sex and age. (a)** Sex-stratified subgroup analysis in the internal validation cohort. **(b)** Age-stratified subgroup analysis in the internal validation cohort. **(c)** Sex-stratified subgroup analysis in the external validation cohort. **(d)** Age-stratified subgroup analysis in the external validation cohort.
